# Geographic variation in baseline innate immune function does not follow variation in aridity along a tropical environmental gradient

**DOI:** 10.1038/s41598-020-62806-1

**Published:** 2020-04-03

**Authors:** Chima J. Nwaogu, Will Cresswell, B. Irene Tieleman

**Affiliations:** 1 Groningen Institute for Evolutionary Life Sciences, University of Groningen, P.O. Box 11103, 9700 CC Groningen, The Netherlands; 2 School of Biology, University of St Andrews, Harold Mitchell Building, St Andrews Fife, KY16 9TH St. Andrews, UK; 3 A.P. Leventis Ornithological Research Institute, Jos, Nigeria; 4 Present Address: Fitzpatrick Institute of African Ornithology, University of Cape Town, 7701 Rondebosch Cape Town, South Africa

**Keywords:** Innate immunity, Ecophysiology

## Abstract

Geographic variation in aridity determines environmental productivity patterns, including large-scale variability in pathogens, vectors and associated diseases. If disease risk decreases with increasing aridity and is matched by immune defense, we predict a decrease in innate immune function along a gradient of increasing aridity from the cool-wet forest to the hot-dry Sahel, from south to north in Nigeria. We sampled blood and measured five innate immune indices from 286 Common Bulbuls *Pycnonotus barbatus* between 6 and 13°N. We sampled in the dry season; we resampled the first location (Jos) also as the last sample location to test temporal change in immune function. Immune indices did not decrease with aridity. One immune index, nitric oxide concentration showed a weak quadratic pattern. In Jos, ovotransferrin concentration, haemagglutination and haemolysis titres increased 12 weeks into the dry season, contrary to expectations that immune indices should decrease with increased dryness. In this tropical system, innate immune function does not decrease with increasing aridity but temporal factors within a location may influence immune function more strongly than spatial variation in aridity, suggesting that immune variation does not follow a simple environmental productivity pattern. Consequently, caution should probably be exercised in predicting effects of climate variability on immune function or disease risk.

## Introduction

Geographic variation in aridity determines environmental productivity patterns^1,2^, including large-scale differences in infectious diseases^3,4^ and/or important vectors for disease transmission^5–7^. Consequently, animal distributions are hypothesized to be adapted to variation in disease risk^8–10^. These patterns are largely driven by spatio-temporal variability in temperature and precipitation, and they underpin the mechanisms that together or independently shape interactions among infectious agents, vectors and hosts, including host contact rate, susceptibility, infectiousness and immunity^11–13^. Assuming that innate immunity - the first line of defense against infection^14^, varies with the risk of infection^15^, we might expect immune investment to decrease with decreasing environmental productivity (or increasing aridity) due to reduced immune challenge^15^ or limited resource availability for immune function^16^ under similar life history circumstances.

Both free living and parasitic species decrease with environmental productivity, and are more diverse in the tropics^7,17–19^. This high biological diversity in tropical environments is not only associated with high environmental productivity, but also with the diversity of environmental conditions within the tropics. But despite the understanding that tropical environments are relatively disease prone and capable of imposing higher immune costs compared with temperate environments^10,20^, the relationship between environmental variability and immune function has rarely been investigated empirically within tropical systems (but see^21^). Such investigations are important for understanding whether immune investment and infection risk influence habitat use in tropical systems and how the life histories of tropical animals may be affected by climate and land use changes^22^.

The environment, however, affects several factors which may affect immune function differently, so it is important to identify how specific environmental factors relate to immune variation in natural systems with clearly variable environmental patterns. Food availability, diet and life history patterns may also vary with environmental productivity in space and time, and these may affect immune function^23,24^. Populations may however, be locally adapted or genetically variable across a species’ range due to limited gene flow, and so populations may respond differently to variation in disease risk, immune challenge or resource constraints^25–27^. Such effects of environment versus genetic differences on life history traits, including immune function, have been investigated over large spatial environmental gradients^17,26,28–30^ and across the annual cycle of temperate animals^31,32^. Overall, results suggest that variation in some immune indices have a genetic background, while others are flexible or rigid to environmental conditions^26^.

**Figure 1:**
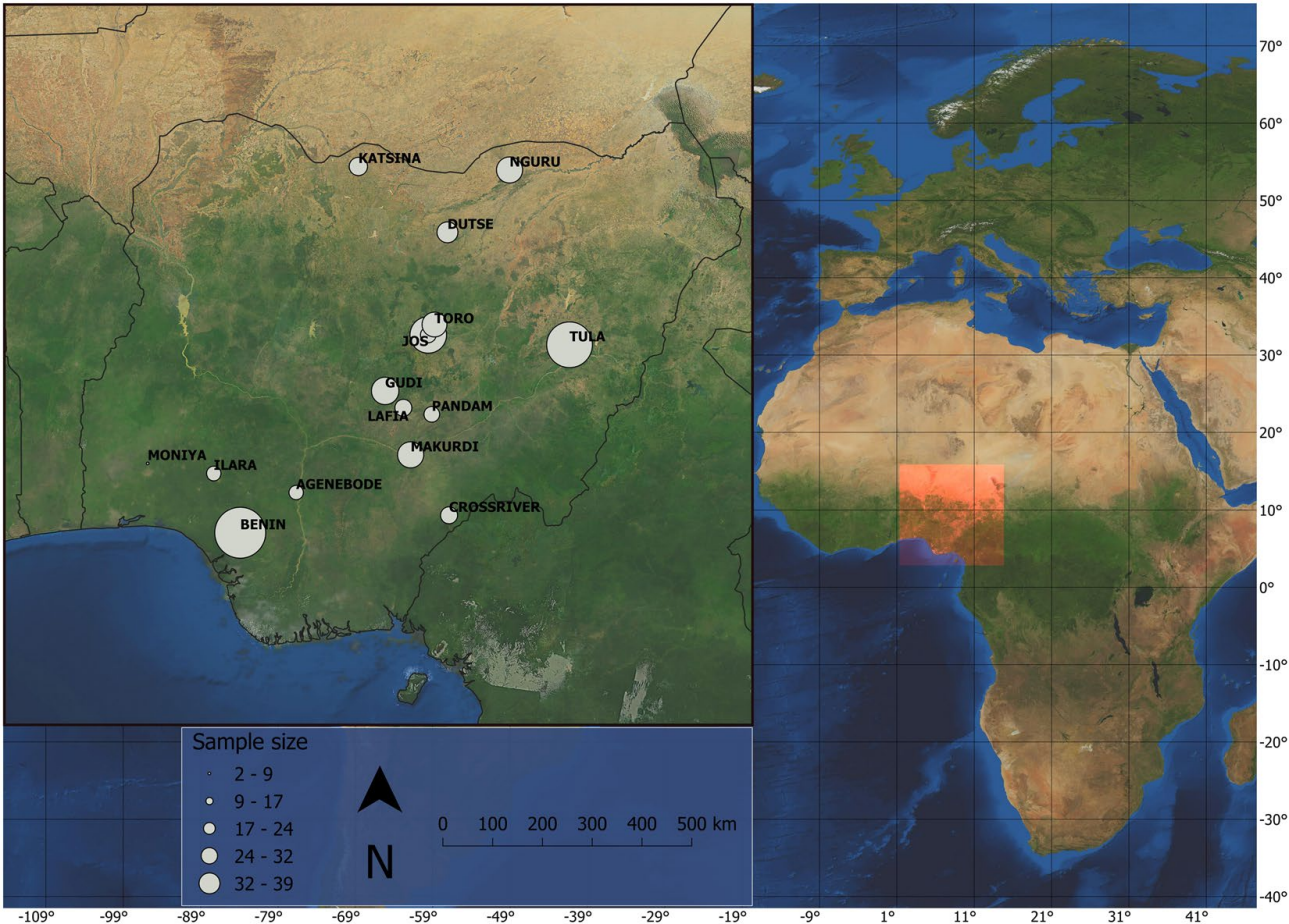
North to south Nigeria represents an important tropical aridity gradient. Map shows study area with 15 localities along an aridity gradient in Nigeria where Common Bulbuls *Pycnonotus barbatus* were sampled at the end of the dry season between 17^th^ January and 8^th^ April 2017. Order in which sites where visited and local site features are provided in Table 1. Point size indicate relative sample size among localities. Map of study area was created using QGIS 3.4: QGIS Development Team (2019). QGIS Geographic Information System. Open Source Geospatial Foundation Project. http://qgis.osgeo.org. GeoJSON Maps of the globe was downloaded from https://geojson-maps.ash.ms/ for country boundaries. Esri. “World Imagery”, “World Imagery Basemap”, 12 Feb 2009, https://www.arcgis.com/home/item.html?id=10df2279f9684e4a9f6a7f08febac2a9 (23/01/2020).

The heterogeneity of environmental conditions in tropical systems allows for a test of predicted global patterns of immune variation in accordance with the expected variation in disease risk^3^. For example, environmental conditions, from the Sahara Desert in the north of West Africa to the coast of the Atlantic Ocean in the south, represent a gradient of decreasing environmental aridity and temperature (Fig. 1). Within Nigeria, locations separated by less than 800 km may differ by over 1600 mm of rain annually. Nigeria experiences a single period of rainfall and one of drought each year, but the amount of rain and duration of the wet season varies latitudinally - the wet season is later and shorter going from south to north. This feature creates a north-south spatial aridity gradient which is most pronounced at the end of the dry season, and a temporal aridity gradient from the onset of the dry season to its end. This aridity gradient may drive spatio-temporal differences in food availability and/or infection risk and thus, may strongly influence life history traits, including immunity.

Some West African bird species are widespread residents across different locations^33–35^. For example, the Common Bulbul *Pycnonotus barbatus* will experience different environmental conditions across their range. Common Bulbuls vary significantly in body size in relation to variation in temperature and aridity^36,37^, with birds from cooler and wetter areas being smaller in body size compared to individuals from more arid locations. They moult in the wet season, but may breed year-round^38^. Across their range they feed on fruits, insects, nectar and seeds depending on availability^39,40^. Such a species is therefore ideal for assessing the relationship between environmental aridity and immune function, assuming similar habitat-use but variable infection risk and food availability across an environmental gradient.

There are no empirical data showing variation in specific pathogen types across Nigeria, and despite increased interest in wild immunology, interpreting variation in immune indices in relation to infection in wild animals is still ambiguous^41–43^. Yet, biomarkers of inflammation respond in different directions upon infection, while natural antibody and complement activity reflect capacity to destroy foreign cells^44,45^. So, simultaneously considering multiple immune indices can improve interpretation when assessing the immune defence status of an animal^46^. Because higher environmental productivity is associated with the prevalence of infectious agents, including bacteria, fungi and vector and water-borne parasites^47,48^, immune indices should decrease with increasing aridity. Increase in biomarkers of inflammation such as haptoglobin, ovotransferrin and nitric oxide concentrations, and natural antibody and complement activity may reflect anticipation of, or response to infection. High immune indices may also reflect absence of food limitation in more productive environments^49^. For example, we know that well fed Common Bulbuls in good body condition maintain high natural antibody and complement activity, but low haptoglobin concentration, whereas poorly fed Bulbuls in poor body condition show the reverse^50^. So, if increasing aridity decreases infection risk, reduced susceptibility to infection should lead lower immune indices with increasing aridity.

Here, we test the hypothesis that baseline immune function decreases with increasing aridity due to expected lower immune challenge and corresponding reduced immune investment due to limited resource availability in more arid environmental conditions. However, because site temperatures during sampling may affect immune function^51^ independent of long-term environmental aridity, we also considered the effect of average site temperature on immune indices. We sampled Common Bulbuls in the dry season, predicting that: (i) immune indices will decrease with increasing aridity from the cool-wet south to the hot-dry north of Nigeria, (ii) immune indices will be lower later in the dry season than earlier in the dry season within a single location.

## Methods

### Ethics statement

Sampling protocol adhered to the local laws of Nigeria and was approved by the A. P. Leventis Ornithological Research Institute’s (APLORI) scientific committee. Methods were carried out in accordance with relevant guidelines and regulation; all birds were handled by a licenced ringer (CJN), blood samples taken were < 10 microliter per gram of a bird and checked and released immediately after processing.

### Study species

Common Bulbuls are sexually monomorphic passerines which are 9–11 cm in body length and weigh 25–50 g. In Table 1, we present detailed information on study populations and capture locations where we sampled Common Bulbuls between 17 January and 8 April 2017.

**Table 1:** Description of 15 sampling locations along the environmental gradient north to south of Nigeria (Fig. 1) where Common Bulbuls *Pycnonotus barbatus* where observed and sampled in the dry season between 17^th^ January and 8^th^ April 2017.

LOCATION	N	Sampling dates	Latitude (°N)	Longitude (°E)	Elevation (m)	Aridity score	% Breeding	% Moulting	Habitat description	Foraging substrate
BENIN	39	Jan. 31–Feb. 1	6.31	5.58	105.66	56.56	83.3	75.0	Forest clearing,	Insects from cassava farm,
									cassava plantation,	pawpaw fruits
									human settlement,	
									fruit gardens	
CROSSRIVER	13	Feb. 21 - Feb. 22	6.62	9.35	264.01	53.09	7.1	7.1	Forest clearing,	Insects from cassava farm,
									Riparian forest fragments,	Ficus fruits,
									cassava plantation	*Alchonia cordifolia* fruits
AGENEBODE	11	Feb. 3 – Feb. 6	7.03	6.59	49	36.19	0	18.1	Farm settlement,	Insects from cashew flowers,
									cashew plantation,	*Alchonia cordifolia* fruits,
									oil palm plantation,	cashew fruits,
									citrus plantation	oil palm fruits
ILARA	11	Feb. 10- Feb. 11	7.37	5.1	368.28	39.66	45.5	90.9	Cassava plantation,	Insects from cashew flowers,
									plantain plantation,	pawpaw fruits,
									fruit gardens,	oil palm fruits
									oil palm plantation	
MONIYA	2	Feb. 8 - Feb. 9	7.55	3.91	232.17	34.48	0	50.0	Forest clearing,	Insects from cassava farm,
									fruit gardens	Ficus fruits
MAKURDI	20	Feb. 24- Feb. 25	7.71	8.66	107.46	35.04	0	0.0	Farm settlement,	Insects from cashew
									forest clearing,	flowers,
									cashew plantation,	pawpaw and
									cassava plantation	Ficus fruits
PANDAM	12	Feb. 16- Feb. 17	8.44	9.04	183	33.26	0	0.0	Dry riverbank,	Ficus fruits
									savannah woodland	
LAFIA	13	Feb. 18- Feb. 19	8.56	8.53	144.14	35.61	23.1	0.0	Human settlement,	Insects from cashew and
									Neem plantation, riparian forest,	Parkia biglobosa flowers,
									fragments, rice farm	neem fruits,ficus fruits
GUDI	21	Feb. 27- Feb. 28	8.86	8.2	422.59	40.77	14.3	0.0	Dense woody vegetation,	Ficus fruits
									Inselbergs,	
									concrete water reservoir	
TULA	35	Mar. 14- Mar. 15	9.7	11.53	356.87	25.27	0	11.4	Mango plantation,	Insects,
									riparian forest fragments,	Mango fruits,
									savannah woodland,	*Syzygium guineense* fruits,
									inselbergs	*Alchonia cordifolia* fruits
										ficus fruits
JOS	28	Jan. 17 – Jan. 19	9.88	8.98	1325	40.49	0	0.0	Riparian forest fragments,	Insects,
									savannah woodland,	*Harungana madagascariensis* fruits
									inselbergs	*Rhus natalensis* fruits
JOSII	12	Apr. 4 – Apr. 8	9.88	8.98	1325	40.49	25	16.6	Riparian forest fragments,	Insects,
									Savannah woodland,	*Rhus natalensis* fuits
									inselbergs	*Jasminum dichotomum* fruits
TORO	19	Mar. 17- Mar. 19	10.06	9.09	929.35	36.11	22.2	5.5	Riparian forest fragments,	Insects from cashew flowers,
									vegetable farms	*Alchonia cordifolia* fruits,
										ficus fruits.
DUTSE	16	Mar. 21– Mar. 23	11.73	9.33	457.94	20.57	12.5	6.3	Date plantation,	Insects from mango trees,
									mango plantation	mango fruits
NGURU	20	Mar. 25 - Mar. 26	12.86	10.45	375.13	12.14	15	10.0	Irrigated farmlands,	Insects from cashew
									neem plantation,	flowers, neem fruits,
									human settlement	cashew fruits
KATSINA	14	Mar. 28 – Mar. 30	12.92	7.72	447.53	17.02	35.7	0.0	Irrigated farmlands,	Insect from onion farm,
									neem plantation	neem fruits

Common Bulbuls were sampled at Jos at the start and end of the study.

### Bird sampling and environmental variables

We travelled between latitude 6 and 13° N (c. 750 km horizontal distance) in Nigeria and mist-netted 286 Common Bulbuls from 15 locations (Fig. 1). Common Bulbuls are resident year-round, so we expect populations to be distinct. We visited each location, including resampling of the first location visited (Jos), in the dry season. Jos is located about mid-way along the aridity gradient and five localities from the most northern sampling location. It was sampled at the start and end of the study, at a 12 weeks interval within the dry season, to test for temporal difference in immune function within a single location. The entire study was carried out within 12 weeks, and all before the first rain in Jos. Apart from Jos, which was sampled at the start and end of the study, we sampled from the southernmost location (Benin) and advanced northward. The sampling pattern (south to north) was designed to avoid sampling any location after the start of the rains since the rains commence earlier in the south, and because we predicted that immune function will decrease with increasing aridity. Otherwise, sampling southern locations after the start of the rains will make any conclusions about the effect of environmental aridity on immune function invalid. By sampling south to north consistent with the onset of the rains, we were close to the start of the wet season in each location during our visit.

All birds were caught using mist-nets and sampled between 6:00 and 11:00 hours. We collected c.300 microliter of blood from each bird into heparinised micro capillary tubes after puncturing the brachial vein with a needle. On average, birds were bled 17.5 ± 8.6 minutes after capture. Samples were stored on ice in the field until processing to separate plasma from cellular fractions, then stored frozen at −20 °C until immune assays were carried out.

We weighed (±0.1 g, Ohaus Scout), and determined occurrence of moult and breeding from each bird in order to account for their possible effects on immune function. Breeding status could only be determined for female birds because males do not carry brood patches and Common Bulbuls are sexually monomorphic.

To assess the relationship between environmental aridity and immune function, we extracted bioclimatic variables for each capture location (Table 1), including annual precipitation, precipitation seasonality, precipitation of the driest quarter, mean annual temperature, temperature seasonality and temperature of the driest quarter from http://www.worldclim.org/bioclim, using the ‘maptools’ and ‘raster’ packages in R. We used the bioclimatic variables extracted to calculate De Martonne aridity index^52^ as a measure of long term environmental aridity in each location: De Martonne aridity index = annual precipitation (mm) / (mean annual temperature (°C) + 10). Locations with values less than 10 are classified as arid, while those with values greater than 40 are classified as humid. Then to assess the possible effect of current weather condition on immune function, we estimated the average site temperature (°C) at mid-day at each capture location during the sampling period using dry bulb temperature records from a hand-held thermometer. We did not consider variation in rainfall because there was no rain at any time during sampling.

### Immune assays

We measured haptoglobin, nitric oxide and ovotransferrin concentration, and haemagglutination and haemolysis titres as indices of innate immune function. Haptoglobin, a positive acute phase protein, and ovotransferrin, a negative acute phase protein both circulate in low baseline concentrations. They function by binding to and removing haem from circulation during infection, making haem unavailable to pathogens^53^. Haptoglobin and ovotransferring initially increase with inflammation^54,55^ (but see^56^), but ovotransferrin concentrations may decrease during very high inflammation because temporarily high free hormones may bind to it. In addition, during infection the liver may produce more haptoglobin at the expense of ovotransferrin^54,57,58^, and this may result in a positive response of haptoglobin and a negative response of ovotransferrin. Multifunctional signalling molecules such as nitric oxide, modulate inflammatory processes but it also participates in the direct killing of parasites and tumor cells^59^, and so should increase with infection. On the other hand, natural antibodies and complementary activities, as quantified by agglutination and haemolysis titre of plasma, form the first line of defense and a useful link between innate and adaptive immunity^60–63^. Natural antibodies function as recognition molecules capable of opsonizing invading microbes and initiating a complement enzyme cascade, which destroys invading pathogens^64,65^.

We quantified plasma haptoglobin concentration (mg/ml) using a functional colorimetric assay which quantifies the haeme-binding capacity of plasma. We followed instructions for the ‘manual method’ provided with a commercially available assay kit (Cat. No.: TP801; Tridelta Development Ltd, Maynooth, Co. Kildere, Ireland)^55^. We calculated within-plate variability for haptoglobin pool, used as standard (n = 4 plates, maximum CV = 0.24, minimum CV = 0.02, mean CV = 0.08) and among-plate variability (n = 8 samples, CV = 0.12) to assess assay consistency.

We quantified plasma nitric oxide (µM) concentration by a colorimetric assay^59^. The method estimates the concentration of nitrate and nitrite in plasma after reducing all nitrates to nitrites using copper-coated cadmium granules. A measurable colour development proportionate to the nitric oxide concentration follows reaction with Griess reagent, and its absorbance is measured by colorimetry. We calculated within-assay variability of chicken plasma, used as standard (n = 8 plates, maximum CV = 0.76, minimum CV = 0.01, mean CV = 0.17) and among-assay variability (n = 16 samples, CV = 0.14) to assess assay consistency.

We quantified ovotransferrin (mg/ml) by estimating the maximum amount of iron required to saturate all ovotransferrin in a sample. We followed a three step process^53^: saturation of ovotransferrin with ferric iron under alkaline conditions, reduction of excess unbound iron by ascorbic acid, then dissociation of ovotransferrin-iron complex under acidic conditions, leading to a colour development whose absorbance is measured by colorimetry. We calculated within-assay variability of chicken plasma, used as standard (n = 10 plates, maximum CV = 1.06, minimum CV = 0.17, mean CV = 0.58) and among-assay variability (n = 20 samples, CV = 1.48) to assess assay consistency.

We quantified natural antibody-mediated haemagglutination and complement-mediated haemolysis titres of plasma samples against 1% rabbit red blood cells (Envigo RMS (UK) Ltd.) in phosphate buffered saline^45^. Both haemagglutination and haemolysis titres were recorded as the number of serial dilution steps in which each function was still observable using an existing rubric^45^. We calculated within-assay variability of chicken plasma used as standard (n = 92 plates, haemagglutination: maximum CV = 0.61, minimum CV = 0, mean CV = 0.09; haemolysis: maximum CV = 1.41, minimum CV = 0, mean CV = 0.36) and among-assay variability (n = 184 samples, haemagglutination: CV = 0.14; haemolysis: CV = 0.56) to assess assay consistency.

We randomised samples before all assays. All colorimetric assays (haptoglobin, nitric oxide and ovotransferrin concentrations) were carried out using the Versamax plate reader (Molecular Devices Sunnyvale, California, US).

### Data analyses

First, we built general linear mixed-effect models to test how aridity index and average site temperature (°C) predict haptoglobin (mg/ml), nitric oxide (µM) and ovotranferrin (mg/ml) concentrations, and haemagglutination and haemolysis titres. Haemolysis titre was log-transformed to achieve normality. We included capture location as a random factor to account for differences between capture locations which may be unrelated to aridity or temperature and to account for the use of common site measures (aridity and temperature) to predict variation in the immune function in multiple birds sampled within a location. We also included body mass and extent of moult, calculated as the proportion of feather mass already regrown^66^ as predictor variables because individual condition and energy expenditure on moulting may affect immune function^67^, and also tested whether body mass(g) varied with aridity after accounting for variation in wing length. We have used wing length previously as index of body size in Common Bulbuls^37^, because wing length is more precisely and more often measured than tarsus length^68^, but using tarsus instead of wing length did not alter results. We did not include breeding status in our models because only females can be scored for breeding using brood patch occurrence. To test whether capture location contributed significantly to variation in immune function i.e. whether immune function differed among capture locations, we built general linear models with similar variables to those included in the general linear mixed-effect models, but without the random factor, capture location. We compared model pairs using analysis of variance to determine whether the inclusion of capture location as random factor improved the explanatory power of the models. In addition, we calculated within and among location coefficient of variation for each immune index (Table S1) and correlation between immune indices (Table S2).

Secondly, to test whether immune indices were lower later in the dry season relative to the start of sampling, over the 12 weeks of the study, we built general linear models to compare the first and second sampling batches in Jos. We included sampling batch as a two-level factor, and body mass as a covariate to account for variation in individual condition.

All statistical analyses were performed in R 3.5.1^69^.

## Results

**Figure 2:**
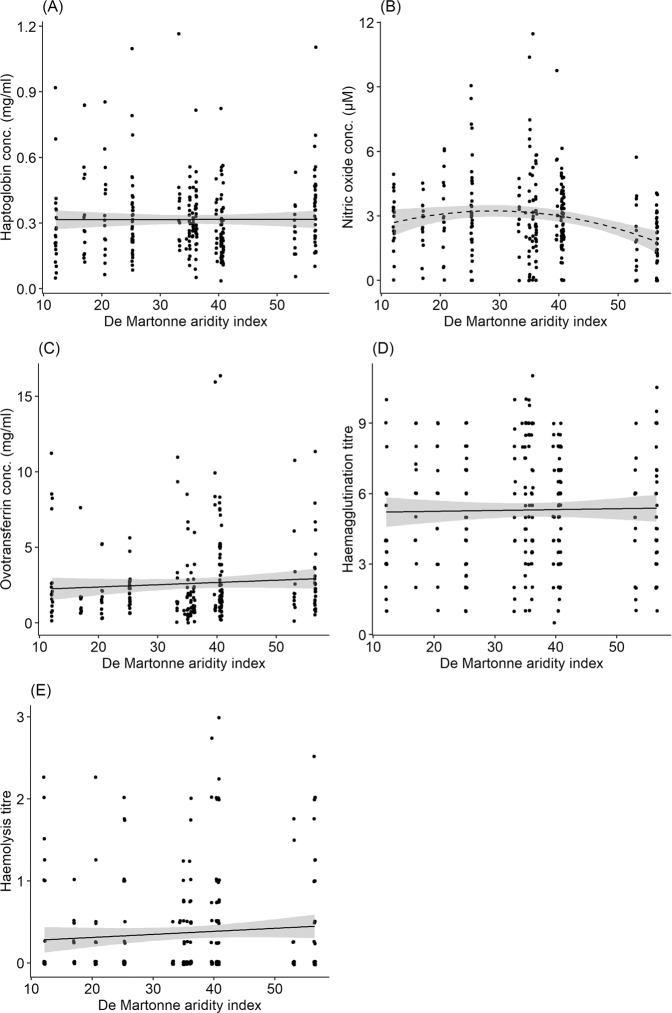
Relationship between (**A**) haptoglobin concentration (mg/ml), (**B**) nitric oxide concentration (µM), (**C**) ovotransferrin concentration (mg/ml), (**D**) haemagglutination titre and (**E**) haemolysis titre in Common Bulbuls and variation in aridity (De Martonne aridity index – high values indicate lower aridity i.e. increased humidity) along an environmental gradient from north to south of Nigeria. Significant correlation highlighted in broken lines.

**Figure 3:**
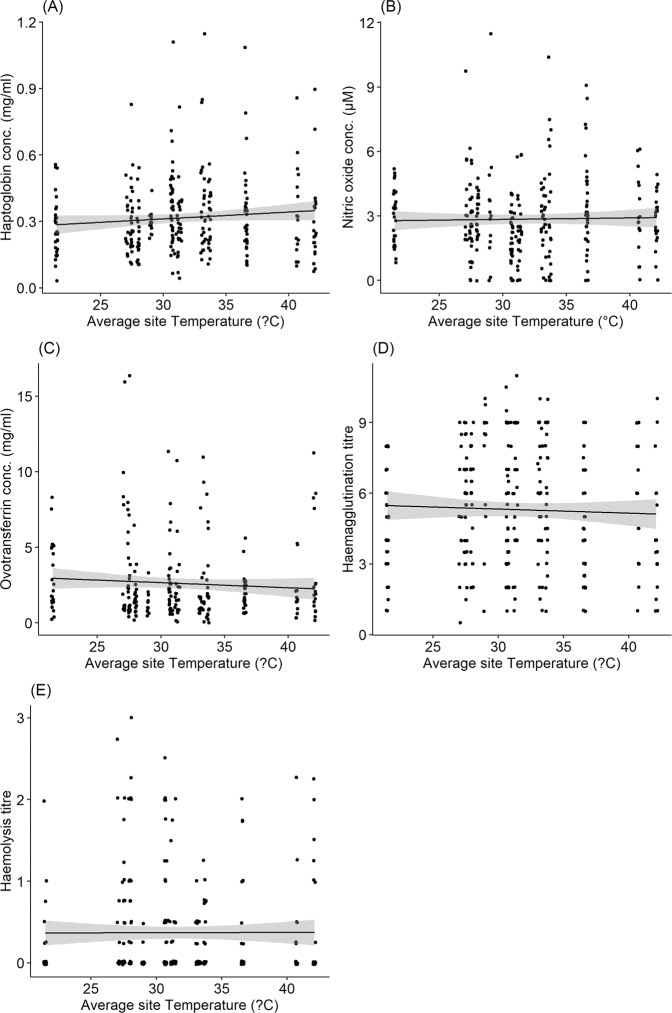
Relationship between (**A**) haptoglobin concentration (mg/ml), (**B**) nitric oxide concentration (µM), (**C**) ovotransferrin concentration (mg/ml), (**D**) haemagglutination titre and (**E**) haemolysis titre in Common Bulbuls and local temperature during sampling along an environmental gradient from north to south of Nigeria.

Immune indices - haptoglobin concentration, ovotranferrin concentration, haemagglutination titre and haemolysis titre - did not vary with aridity along the environmental gradient (Fig. 2, Table 2). Nitric oxide concentration however, showed a weak quadratic pattern, first decreasing with aridity along the gradient and then increasing subsequently (Fig. 2, Table 2). Haptoglobin concentration, nitric oxide concentration, ovotransferrin concentration, haemagglutination titre and haemolysis titre did not vary with local temperature during the sampling period (Fig. 3, Table 2). Body mass explained variation in haemagglutination titre but not haptoglobin, nitric oxide and ovotransferrin concentration, and haemolysis titre (Table 2). Body mass (corrected for wing length), decreased significantly with increasing aridity (aridity: χ^2^_1, 283_  =  9.76, P  =  0.002, wing length: χ^2^_1, 283_  =  262.62, P  <  0.001). Sampling location explained a significant proportion of the variation in haemagglutination titre (Va = 0.61 ± 0.78, P = 0.03) and haemolysis titre (Va = 0.01 ± 0.10, P = 0.04), but not in haptoglobin concentration (Va = 0.001 ± 0.02, P = 1), nitric oxide concentration (Va = 0.07 ± 0.26, P = 0.85) and ovotransferrin concentration (Va = 0.91 ± 0.95, P = 0.08). The extent of primary moult did not explain variation in any of the immune indices. Individual bioclimatic variables did not correlate with immune indices (see Fig. S1–5). The only exceptions were precipitation of driest quarter which predicted nitric oxide concentration and mean annual temperature which predicted ovotransferrin concentration (Table S3).

**Table 2:** Summary of variation in immune indices of Common Bulbuls *Pycnonotus barbatus* sampled across 15 locations along an aridity gradient (‘Gradient’) in Nigeria (Fig. 1) or on two occasions at Jos (‘Jos’).

	Variable	Df	Haptoglobin		Nitric oxide		Ovotransferrin		Haemagglutination		Haemolysis	
			Chisq	P	Chisq	P	Chisq	P	Chisq	P	Chisq	P
Gradient	Aridity index	1	0.28	0.60	**4.81**	0.03*	0.04	0.83	0.01	0.92	0.42	0.51
	Aridity index^2^	1			**8.53**	<0.01**						
	Temperature	1	1.86	0.17	0.05	0.82	0.20	0.65	0.06	0.81	0.25	0.62
	Body mass	1	0.02	0.88	0.13	0.72	0.11	0.74	**5.34**	0.02*	2.40	0.12
	Moult extent	1	0.92	0.34	1.15	0.28	1.50	0.22	0.65	0.42	1.16	0.28
	**Variable**	**Df**	**F**	**P**	**F**	**P**	**F**	**P**	**F**	**P**	**F**	**P**
Jos	Batch	1	0.14	0.71	0.08	0.78	**5.01**	0.03*	**7.99**	0.01**	3.27	0.08
	Body mass	1	0.00	0.98	1.80	0.19	0.89	0.35	0.09	0.76	0.15	0.70

Body mass, extent of moult and temperature during period of sampling were included in all models to test the possible effects of body condition, energy expenditure on moulting and environmental condition during capture. A quadratic term for aridity index was included in the model for nitric oxide concentration. Significant effects are highlighted in bold.

**Figure 4:**
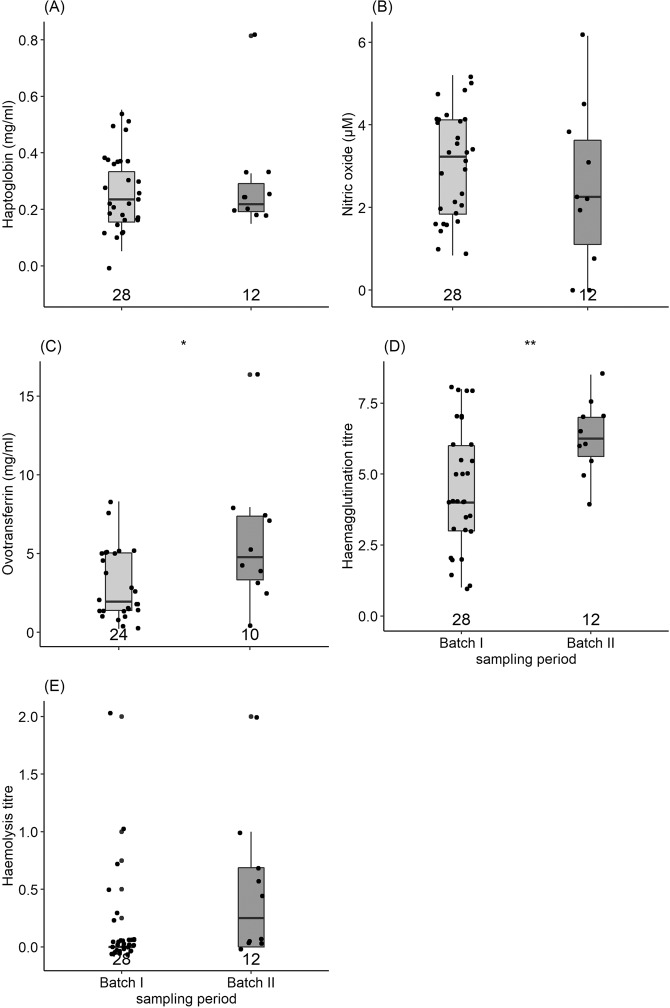
Difference in (**A**) haptoglobin concentration (mg/ml), (**B**) nitric oxide concentration (µM), (**C**) ovotransferrin concentration (mg/ml), (**D**) haemagglutination titre and (**E**) haemolysis between Common Bulbuls sampled about mid-way along an aridity gradient south to north of Nigeria (Fig. 1) at 12 weeks interval in the dry season, corresponding to start and end of the sampling period along the gradient. Sample sizes are indicated at the base of each plot. *Indicate significant difference.

Ovotransferrin concentration and haemagglutination titre were significantly higher, and haemolysis titre was marginally insignificantly higher during the second sampling batch in Jos, 12 weeks further in the dry season (Fig. 4, Table 2). Haptoglobin and nitric oxide concentrations did not differ between sampling batches in Jos (Fig. 4, Table 2).

## Discussion

We tested the hypothesis that immune function decreases with increasing aridity along an environmental gradient in Nigeria and found no clear evidence in support of this hypothesis. We observed however, that nitric oxide concentration first decreased, before increasing with increasing aridity from south to north. Within the single site, Jos, ovotransferrin concentration and haemagglutination increased significantly while haemolysis titre increased marginally 12 weeks later in the dry season, when environmental conditions were expected to be more arid. However, although immune indices were largely as variable within as among locations (Table S1), variation in haemagglutination and haemolysis titres were partly explained by capture locations, independent of aridity. The lack of a decrease in immune indices with increasing aridity was unexpected^17,19,30^, but suggests, in this system, that variation in innate immune function does not follow a simple environmental productivity pattern. We discuss the implications of these findings and present alternative explanations for the lack of correlation between immune function and aridity.

The hypothesis that immune function might be attenuated in arid conditions is based on the assumption that disease risk decreases with increasing aridity^15,29,30,70^ and that immune investments are lower during periods of lower disease risk or reduced resource availability^71^. Although we did not quantify infection risk and food availability, lower aridity is associated with higher environmental productivity, and should support higher pathogen and food abundance. If the assumption that pathogen pressure decreases with increasing environmental aridity holds in this system, then it is not clear why immune indices did not decrease with increasing aridity. However, individual factors such as infection, physiological state and personality^72,73^ may provoke larger within, than among, population variability, thus, obscuring the relationship between environmental factors and immune function. The high among-individual variability observed within localities in this study may be due to such intrinsic individual differences. Variation in innate immune function may therefore not reflect abundance of antigens in the environment^74^, immune function may be more related to actual infection on individuals, and this may depend on habitat use^75^ and annual cycle stage. Another factor that may affect immune function is diet^24,50,76^, and Common Bulbuls feed predominantly on fruits and insects despite spatial differences in aridity (Table 1). All individuals may, therefore, maintain similar levels of innate immune function because their diet was similar across the aridity gradient, even if food was limited for some individuals. These interactions between individual and environmental factors may partly explain contrasting results in studies exploring latitudinal and regional patterns in immune indices. For future studies, estimating parasitic infection in addition to environmental ‘immunobiome’ pressure^15^ along a gradient of interest should then provide better understanding of variation in disease risk.

Temporal variation in environmental conditions within a single location may be a more important source of variation in immune function than spatial variation in aridity. Three of the five immune indices we measured were higher 12 weeks later at the same site (Jos). The reason for these temporal differences is not obvious, but during the second batch of sampling at Jos, local temperatures were c. 6 °C higher, and 25.0% and 16.6% of birds were breeding and moulting respectively, compared to none breeding or moulting during the first period of sampling (Table 1). Although these temporal differences in some immune indices were significant, they do not suggest, however, an attenuation of immune function with increased aridity^30^ or occurrence of annual cycle stages^77^. Instead they suggest flexibility within individuals to respond to local conditions. This result is consistent with findings from our earlier year-round study on Common Bulbuls in Jos where we found higher immune indices in the dry season compared to the wet season, and a further increase in some immune indices as the dry season progressed^43^. The high variability within locations (Table S1) confirms that innate immunity is highly flexible^26^. A similar sampling effort during the wet season may then help to confirm this hypothesis because the rains may cause a greater degree of change to environmental conditions in more arid northern localities than in humid southern localities that are relatively wet year-round. We would then expect higher immune indices in more arid locations during the wet season if variation in innate immune function is seasonal and results from a response to a change in infection risk relative to the degree of change in environmental conditions between the dry season and the wet season.

The quadratic relationship between nitric oxide concentration and aridity (a decrease followed by an increase with increasing aridity), deviated from the overall pattern we predicted, and from the observed pattern for other immune indices. Studies in poultry have shown increase in nitric oxide production during coccidian infection^24,78,79^, and this is often associated with increased humidity aiding coccidian oocysts sporulation: but we did not assess infection status in this study. In a previous study with Common Bulbuls, nitric oxide concentration was highest around the interface between the wet and dry season^43^. This period coincides with the small breeding peak just before the onset of moult in the species^38^. In the same study, breeding Common Bulbuls had higher nitric oxide concentration, while moulting birds had lower concentrations, and this pattern was consistent within individuals^43^. Common Eiders *Somateria mollissima* also show higher nitric oxide concentration during breeding^80^. Therefore, although nitric oxide is a mediator of inflammatory response and should increase with infection, it is a multifunctional signalling molecule which may vary with the stress and work load^81^ associated with annual cycle stages. In the current study, there was no clear pattern in breeding occurrence along the gradient, but there were more moulting birds in the south compared to the north. Thus, the relative numbers of breeding and moulting birds (See Table 1) may explain the pattern of variation in nitric oxide concentration in different localities.

We did not consider differences in immune indices between sexes because Common Bulbuls are sexually monomorphic. Moreover, in previous studies with Common Bulbuls where we determined sex molecularly, there were no sex related differences for the same immune indices^43,50^. Common Bulbuls are socially monogamous, so we expect a one-to-one sex ratio. Differences between sexes should be unimportant when populations are compared based on randomly sampled birds, except where the effect of aridity on immune function depends on sex.

There is no obvious barrier to gene flow that should lead to population structuring among Common Bulbul populations in Nigeria, however, the species is locally resident and shows a strong clinal body size^37^ and moult pattern along the environmental gradient. Body size and moult patterns suggest local adaptation, and this is consistent with rainfall variation along the gradient. But we cannot say whether the absence of a pattern in immune indices suggests the absence of local adaptation to infection risk or similar infection risk or similar response to different infectious agents. Recent multi-species studies on MHC-I diversity suggest that the acquired immune system of birds has evolved greater pathogen recognition in wetter tropical environments^82^. So, perhaps, the aspects of innate immune function we considered require greater environmental variability to show significant local variation.

Our result raises questions about the specific environmental factors that are responsible for latitudinal variation in infection risk or immune function^9,17^. Except for nitric oxide, none of the other immune indices varied with aridity, average site temperature or the other selected bioclimatic variables (Figs. S1–5). Studies involving larger latitudinal gradients show that mean annual temperature seems to explain variation in parasite load and immune indices better than precipitation^19,83^. Results from studies that used similar immune indices to the ones used in this study are equivocal: a study^30^ on different species of adult larks (Alaudidae) along an aridity gradient spanning desert, temperate and tropics showed negative correlations between aridity and haemagglutination and haemolysis titres, and haptoglobin concentration, but no correlations between aridity and ovotransferrin concentration. However, another study^83^ on antimicrobial proteins in eggs from larks in different environments showed contrasting patterns between ovotransferrin and lysozyme concentration in egg albumen. Elsewhere in the tropics, a study^21^ on chicks of Red-capped Larks *Calandrella cinerea* from three climatically distinct locations in Kenya recorded no differences between immune indices, even though these chicks showed significant differences in growth patterns. Like Kenya, none of the locations from which we sampled Common Bulbuls qualifies as ‘arid’ because none had a De Martonne aridity index^52^ less than 10 and all locations receive above 400 mm of rainfall annually despite seasonal precipitation and temperature variation. Again, perhaps, immune function requires greater environmental variability^29,30^ to show a spatial pattern.

In summary, we can conclude that in this tropical system and within the dry season, innate immune function does not follow a simple environmental productivity pattern, and this may apply to disease risk^8,10,84^. The pattern we observed is unlikely to have arisen from a blurring of the expected pattern by a temporal sampling bias because immune indices did not decrease further in the dry season in Jos, where we sampled twice before and after the other locations. However, large within-location variability due to among-individual variation in life history states, infection rates, or immunity may have obscured associations along the aridity gradient. We therefore highlight the need to empirically test assumptions of variation in disease risk or resource limitation due to variation in environmental conditions. Several studies have used immune indices or pathogen pressure alone to test hypotheses about immune function or disease risk^8,9,20,85–90^ and this may lead to misleading interpretations. More generally, caution should probably be exercised when predicting effects of climate variability on immune function and disease risk, because the relationship between infection risk and environmental conditions may be more complex.

## Supplementary information

Supplementary information.
