# Supplementary material for: Geographic variation in baseline innate immune function does not follow variation in aridity along a tropical environmental gradient
Source: Sci Rep. 2020 Apr 3;10:5909. doi: 10.1038/s41598-020-62806-1 (PMC7125124; doi:10.1038/s41598-020-62806-1)
Supplement: Supplementary file 1 — Supplementary information. [file 41598_2020_62806_MOESM1_ESM.docx]

**Supplementary information**

**Geographic variation in baseline innate immune function does not follow variation in aridity along a tropical environmental gradient**

Chima J. Nwaogu^1, 2, 3*^**^†^**, Will Cresswell^2, 3^ and B. Irene Tieleman^1^

^1^Groningen Institute for Evolutionary Life Sciences, University of Groningen, P.O. Box 11103, 9700 CC, Groningen, The Netherlands.

^2^School of Biology, University of St Andrews, Harold Mitchell Building, St Andrews Fife KY16 9TH, UK.

^3^ A.P. Leventis Ornithological Research Institute, Jos, Nigeria.

*Corresponding author email : [c.j.nwaogu@rug.nl](mailto:c.j.nwaogu@rug.nl)

**^†^**Corresponding author’s current address: Fitzpatrick Institute of African Ornithology, University of Cape Town, 7701, Rondebosch, Cape Town, South Africa.


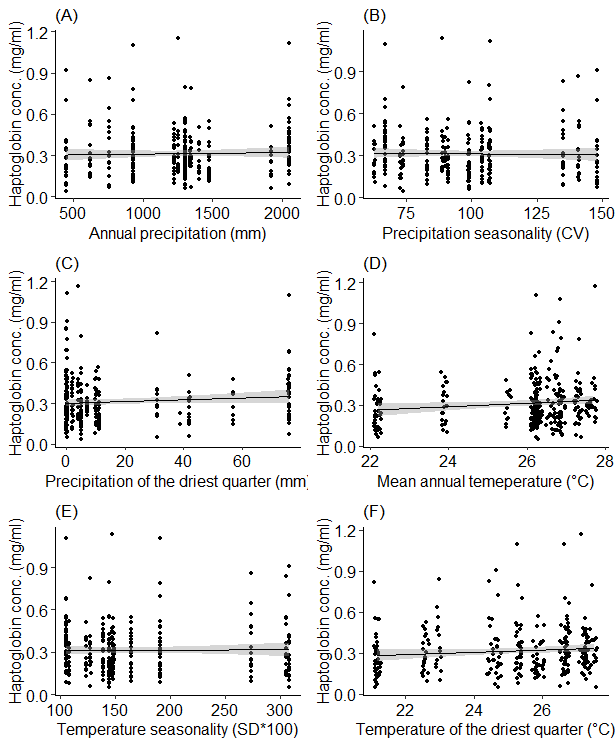


**Figure S1:** Relationship between haptoglobin concentration (mg/ml) in Common Bulbuls *Pycnonotus barbatus* sampled across 15 locations along an environmental gradient in Nigeria and (A) annual precipitation (mm), (B) precipitation seasonality (CV), (C) precipitation of the driest quarter (mm), (D) mean annual temperature (°C), (E) temperature seasonality (SD*100) and (F) temperature of the driest quarter (°C).


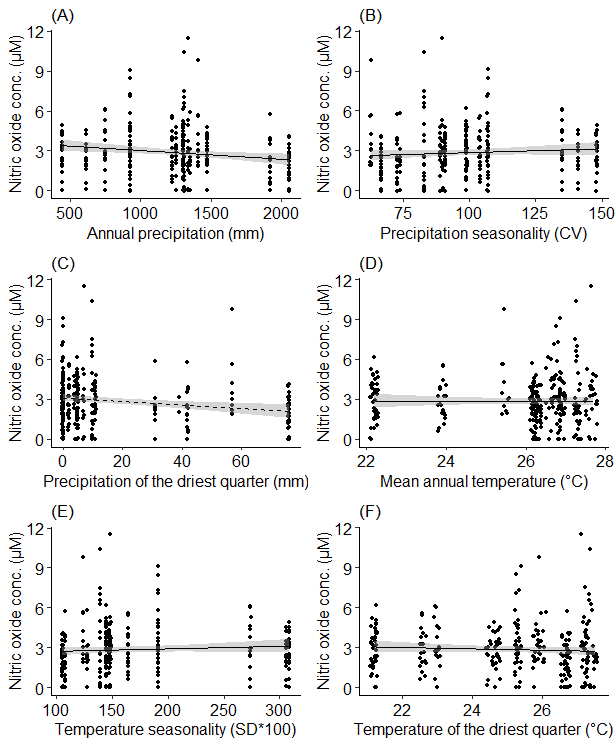


**Figure S2:** Relationship between nitric oxide concentration (µM) in Common Bulbuls *Pycnonotus barbatus* sampled across 15 locations along an environmental gradient in Nigeria and (A) annual precipitation (mm), (B) precipitation seasonality (CV), (C) precipitation of the driest quarter (mm), (D) mean annual temperature (°C), (E) temperature seasonality (SD*100) and (F) temperature of the driest quarter (°C). Significant correlation highlighted in broken lines.


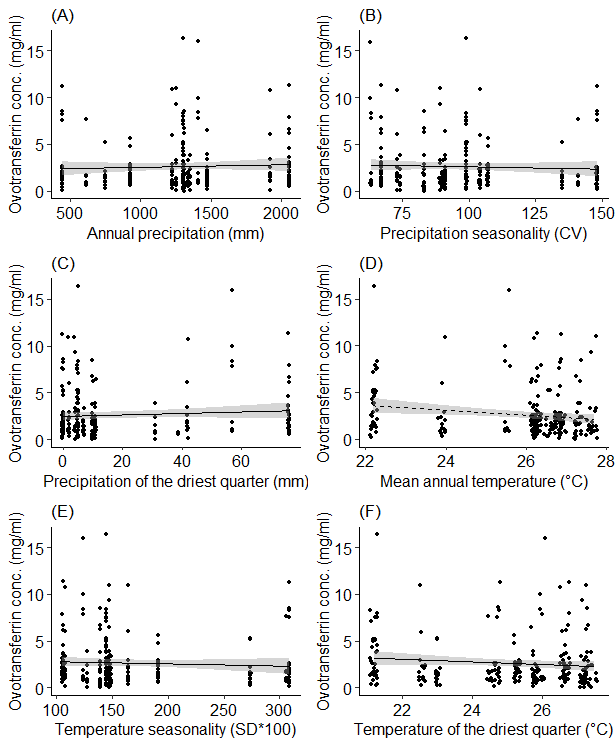


**Figure S3:** Relationship between ovotransferrin concentration (mg/ml) in Common Bulbuls *Pycnonotus barbatus* sampled across 15 locations along an environmental gradient in Nigeria and (A) annual precipitation (mm), (B) precipitation seasonality (CV), (C) precipitation of the driest quarter (mm), (D) mean annual temperature (°C), (E) temperature seasonality (SD*100) and (F) temperature of the driest quarter (°C). Significant correlation highlighted in broken lines.


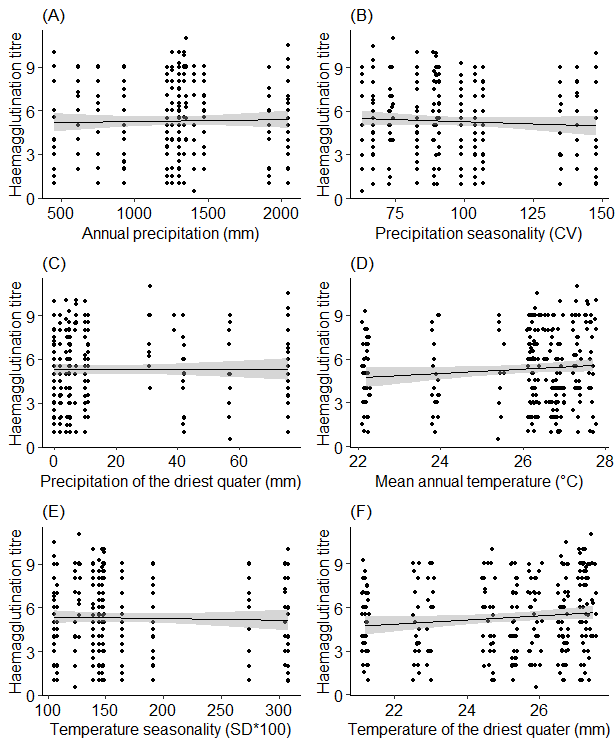


**Figure S4:** Relationship between haemagglutination titre in Common Bulbuls *Pycnonotus barbatus* sampled across 15 locations along an environmental gradient in Nigeria and (A) annual precipitation (mm), (B) precipitation seasonality (CV), (C) precipitation of the driest quarter (mm), (D) mean annual temperature(°C), (E) temperature seasonality (SD*100) and (F) temperature of the driest quarter (°C).


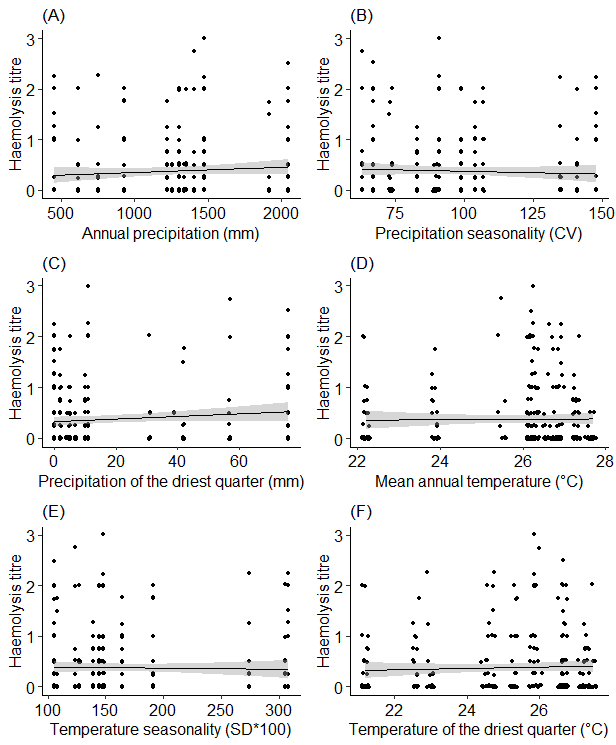


**Figure S5**: Relationship between haemolysis titre in Common Bulbuls *Pycnonotus barbatus* sampled across 15 locations along an environmental gradient in Nigeria and (A) annual precipitation (mm), (B) precipitation seasonality (CV), (C) precipitation of the driest quarter (mm), (D) mean annual temperature (°C), (E) temperature seasonality (SD*100) and (F) temperature of the driest quarter (°C).

**Table S1**: Variation in immune indices of Common Bulbuls *Pycnonotus barbatus* within and among 15 sampling localities along an environmental gradient of varying aridity north to south of Nigeria. The second sampling round in Jos accounts for the 16^th^ location in the table.

| **Assay** | **Variation** | **n** | **Mean** | **Coefficient of variation** | | |
| --- | --- | --- | --- | --- | --- | --- |
|  |  |  |  | **Mean** | **Max** | **Min** |
| **Haptoglobin** | Within-location | 16 locations |  | 0.51 | 0.77 | 0.18 |
|  | Among-location | 285 samples | 0.31 (mg/ml) | 0.55 |  |  |
| **Nitric oxide** | Within-location | 16 locations |  | 0.58 | 0.91 | 0.30 |
|  | Among-location | 285 samples | 2.85 (µM) | 0.64 |  |  |
| **Ovotransferrin** | Within-location | 16 locations |  | 0.87 | 1.17 | 0.24 |
|  | Among-location | 234 samples | 2.63 (mg/ml) | 1.03 |  |  |
| **Haemagglutination** | Within-location | 16 locations |  | 0.44 | 0.58 | 0.08 |
|  | Among-location | 284 samples | 5.29 (titre) | 0.48 |  |  |
| **Haemolysis** | Within-location | 16 locations |  | 1.75 | 3.46 | 0.00 |
|  | Among-location | 284 samples | 0.38 (titre) | 1.69 |  |  |

**Table S2**: Correlation matrix between five innate immune indices of Common Bulbuls *Pycnonotus barbatus* sampled in 15 localities along an environmental gradient of varying aridity north to south of Nigeria.

|  | *Haptoglobin* | *Ovotranferrin* | *Nitric oxide* | *Haemagglutination* | *Haemolysis* |
| --- | --- | --- | --- | --- | --- |
| *Haptoglobin* |  | 0.025 | 0.089 | 0.002 | -0.023 |
| *Ovotranferrin* |  |  | -0.004 | 0.014 | 0.057 |
| *Nitric oxide* |  |  |  | -0.181^**^ | -0.082 |
| *Haemagglutination* |  |  |  |  | 0.018 |
| *Haemolysis* |  |  |  |  |  |
| *Computed correlation used pearson-method with listwise-deletion.* | | | | | |

**Table S3**: Summary test statistics for correlation between immune indices in Common Bulbul *Pycnonotus barbatus* and De Martonne aridity index, local site temperature and selected individual bioclimatic variables - mean annual temperature, annual precipitation, temperature seasonality and precipitation seasonality (Fig. S1 - 5) all extracted from <http://www.worldclim.org/bioclim>.

|  |  | **Haptoglobin** | |  | **Nitric oxide** | |  | **Ovotransferrin** | |  | **Haemagglutination** | |  | **Haemolysis** | |  |
| --- | --- | --- | --- | --- | --- | --- | --- | --- | --- | --- | --- | --- | --- | --- | --- | --- |
| **Variable** | **Df** | **Chisq** | **P** |  | **Chisq** | **P** |  | **Chisq** | **P** |  | **Chisq** | **P** |  | **Chisq** | **P** |  |
| **Annual Precipitation** | 1 | <0.01 | 0.97 |  | 3.70 | 0.05 | . | 0.26 | 0.61 |  | 0.02 | 0.89 |  | 0.30 | 0.58 |  |
| **Precipitation seasonality** | 1 | 0.00 | 0.96 |  | 0.31 | 0.58 |  | 0.23 | 0.63 |  | 0.50 | 0.48 |  | 0.13 | 0.72 |  |
| **Precipitation of the driest quarter** | 1 | 1.07 | 0.3 |  | **9.24** | **<0.01** | ****** | 0.55 | 0.45 |  | 0.001 | 0.99 |  | 1.02 | 0.31 |  |
| **Mean Annual temperature** | 1 | 3.26 | 0.07 | . | <0.01 | 0.97 |  | **4.74** | **0.03** | ***** | 1.20 | 0.27 |  | 0.09 | 0.76 |  |
| **Temperature seasonality** | 1 | 0.14 | 0.71 |  | 0.24 | 0.63 |  | 0.41 | 0.52 |  | 0.13 | 0.72 |  | 0.02 | 0.89 |  |
| **Temperature of the driest quarter** | 1 | 1.62 | 0.2 |  | 0.25 | 0.61 |  | 1.92 | 0.16 |  | 1.81 | 0.18 |  | 0.004 | 0.95 |  |
